# Real-world experience with first-line pembrolizumab plus chemotherapy in Vietnamese patients with stage IV esophageal squamous cell carcinoma

**DOI:** 10.3389/fimmu.2026.1713860

**Published:** 2026-02-25

**Authors:** Thuan Van Tran, Phuong Thu Nguyen, Quang Van Le, Giang Huong T. Nguyen, Huy Le Trinh, Kien Hoang Le, Giap Ngoc Hoang

**Affiliations:** 1 Ministry of Health, Hanoi, Vietnam; 2 Department of Oncology, Hanoi Medical University, Hanoi, Vietnam; 3 Vietnam National Cancer Hospital, Hanoi, Vietnam; 4 Bach Mai Hospital, Hanoi, Vietnam; 5 VNU-MPUH, Linh Dam University of Medicine and Pharmacy Hospital, Vietnam National University, Hanoi, Vietnam

**Keywords:** esophageal squamous cell carcinoma, first-line therapy, pembrolizumab, real-world outcomes, Vietnam

## Abstract

**Introduction:**

Esophageal squamous cell carcinoma (ESCC) remains a major cause of cancer-related mortality in Asia. Pembrolizumab in combination with chemotherapy has become a standard first-line option based on phase III trial data, but real-world evidence from Vietnam is lacking. This study examined the clinical characteristics, disease course, and treatment-related toxicities of Vietnamese patients with stage IV ESCC receiving this regimen.

**Methods:**

We conducted a single-center, single-arm, retrospective analysis of 53 patients with stage IV ESCC treated with pembrolizumab plus platinum- and fluoropyrimidine-based chemotherapy. Information on demographics, clinical presentation, pathology, biomarker status, treatment regimens, patterns of progression or recurrence, and adverse events (AEs) was collected from electronic health records.

**Results:**

The mean age was 59 years, with nearly all patients being male. Most presented with advanced T3/N2 disease and dysphagia at diagnosis. PD-L1 status was unavailable in about one-third of cases. Common chemotherapy backbones were CF (50.9%) and XELOX/SOX (24.5%). Progression or recurrence was documented in 24.5% of patients, and 20.8% occurred within six months. Lymph nodes were the most frequent site of progression, followed by local and distant recurrence. Hematologic toxicities were common, including neutropenia (43.4% all grades, 13.2% grade ≥3) and anemia (50.9% all grades). Nausea (20.8%) and diarrhea (5.7%) were the main gastrointestinal toxicities. Immune-related events included hypothyroidism (3.8%) and pneumonitis (3.8%).

**Conclusions:**

In this Vietnamese cohort, pembrolizumab plus chemotherapy showed a manageable safety profile and recurrence patterns consistent with those reported internationally. These findings add region-specific real-world evidence and underline the importance of broader multi-center studies with longer follow-up to inform practice in advanced ESCC.

## 1 Introduction

Esophageal cancer is a major health problem worldwide, ranking among the most common cancers and a leading cause of cancer mortality. Globally, over 600,000 new cases and more than 540,000 deaths from esophageal cancer were estimated in 2020, with approximately 85% of cases being squamous cell carcinoma (1). Histologically, the disease is divided into esophageal adenocarcinoma and esophageal squamous cell carcinoma (ESCC). While adenocarcinoma predominates in many Western populations, ESCC accounts for the majority of cases in East Asia and in several low- and middle-income countries. Despite therapeutic progress, patients with advanced or metastatic ESCC continue to have poor outcomes; historically, palliative chemotherapy has resulted in a median survival of only around 10 months.

For many years, first-line treatment for advanced ESCC consisted of platinum- and fluoropyrimidine-based regimens, which achieved objective response rates of 30–40% and median progression-free survival of 4–6 months. The introduction of immune checkpoint inhibitors (ICIs) targeting programmed cell death-1 (PD-1) has improved treatment options. Pembrolizumab, a PD-1 monoclonal antibody, showed overall survival benefit in previously treated patients in the KEYNOTE-181 trial, particularly among those with a PD-L1 combined positive score (CPS) ≥ 10, where median OS was 10.0 vs 6.5 months comparing pembrolizumab vs chemotherapy in the Asian ESCC subgroup (2). The phase III KEYNOTE-590 study subsequently demonstrated that pembrolizumab combined with chemotherapy improved overall and progression-free survival compared with chemotherapy alone in the first-line setting; in the Japanese subgroup, median OS was 17.6 vs 11.7 months with pembrolizumab + chemo vs chemo alone (3).

Although pivotal trials have established the benefit of pembrolizumab plus chemotherapy in advanced ESCC, their findings may not fully reflect routine practice, where patients often present with more comorbidities and less access to biomarker testing. In Vietnam, data on real-world use of this regimen are lacking, and patterns of disease presentation, diagnostic availability, and treatment resources may differ from those in other regions. We conducted this study to characterize Vietnamese patients with stage IV ESCC treated with first-line pembrolizumab and chemotherapy, focusing on baseline clinical and pathological features, biomarker status, and the observed patterns of disease progression and treatment-related toxicities in daily practice.

## 2 Materials and methods

### 2.1 Study design and setting

This was a single-center, real-world, single-arm, retrospective-prospective cohort study conducted at the National Cancer Hospital in Hanoi, Vietnam, with a recruitment and follow-up period spanning from January 1, 2024, to January 1, 2025. Ethical approval for the study protocol was obtained from the Institutional Review Board of Hanoi Medical University (Approval No. 1630/GCN-HMUIRB). For the retrospective component, the requirement for individual patient consent was waived due to the use of de-identified patient data retrieved from electronic health records. Conversely, all patients enrolled in the prospective component of the study provided written informed consent prior to participation and retained the right to withdraw at any time.

### 2.2 Study population

The study population comprised a total of 53 patients who met all eligibility criteria for a diagnosis of unresectable locally advanced or metastatic (Stage IV) ESCC and were initiating first-line systemic treatment.

Patients were included if they had a histologically confirmed diagnosis of unresectable locally advanced or metastatic (Stage IV) ESCC, with no indication for curative surgery or radiotherapy. They must have had an Eastern Cooperative Oncology Group (ECOG) performance status of 0–1 and the presence of at least one measurable lesion according to the Response Evaluation Criteria in Solid Tumors (RECIST) version 1.1, which allowed for an assessment of treatment response. In routine clinical practice at our center, first-line pembrolizumab combined with platinum/fluoropyrimidine chemotherapy is generally initiated in patients with adequate functional status; therefore, inclusion was restricted to those with an ECOG performance status of 0–1 to reflect real-world prescribing patterns for this regimen. Baseline ECOG performance status was obtained from the treating oncologist’s documentation at the time of treatment initiation in the electronic medical record, and patients without a clearly documented baseline ECOG score were not included. Additionally, patients must not have received any prior systemic anti-cancer therapy for advanced or metastatic disease. Patients’ full medical records had to be available for inclusion.

Patients were excluded if their ESCC was locally advanced but potentially resectable or treatable with curative radiotherapy. Other exclusion criteria included active autoimmune disease or a history of autoimmune disease requiring systemic immunosuppressive therapy within the past two years, and prior treatment with an immune checkpoint inhibitor such as pembrolizumab or other agents targeting PD-1 or PD-L1. The study also excluded patients with a history of serious infections requiring systemic treatment, significant comorbidities (such as uncontrolled hypertension, uncontrolled diabetes mellitus, or severe cardiac, pulmonary, or renal dysfunction), pregnancy or breastfeeding, severe chronic conditions threatening life, a history of allergy to any of the study medications, or non-compliance with the study protocol or discontinuation of treatment for non-medical reasons.

A comprehensive review of medical records identified patients who met these criteria, resulting in a final cohort of 53 eligible patients for analysis. It is noted that a small number of patients had incomplete data for certain variables, such as chemotherapy regimen and tumor location, which is a known limitation of real-world retrospective data collection.

### 2.3 Data collection

All consecutive patients with stage IV ESCC who initiated pembrolizumab plus chemotherapy during the study period were first identified from institutional treatment records. Eligibility criteria were then verified through a structured review of baseline electronic medical records, including histological confirmation, disease stage, prior systemic therapy for advanced or metastatic disease, the presence of measurable disease, and ECOG performance status.

Clinical information was obtained through a systematic review of electronic health records and patient charts at the National Cancer Hospital. Data were abstracted into a structured case report form by trained researchers, and all entries were subsequently cross-checked to minimize errors and ensure internal consistency. To protect confidentiality, datasets were anonymized and de-identified prior to analysis.

The collected variables covered several domains. Demographic data included age, sex, ethnicity, occupation, smoking habits, and alcohol consumption. Baseline clinical information comprised body weight, height, body mass index (BMI), Eastern Cooperative Oncology Group (ECOG) performance status, history of gastric or other chronic medical conditions, and history of drug allergy.

Disease-related characteristics were extracted from diagnostic evaluations. These encompassed presenting symptoms, the degree of dysphagia, extent of weight loss, endoscopic and imaging findings on tumor location and morphology, histological grading, T and N staging based on the AJCC/UICC 8th edition, and the presence and distribution of distant metastases. Biomarker information included programmed death-ligand 1 (PD-L1) expression, reported as Combined Positive Score (CPS), and mismatch repair deficiency/microsatellite instability-high (dMMR/MSI-H) status where available.

Treatment-related data included the chemotherapy backbone used (CF, FOLFOX, XELOX/SOX), pembrolizumab dosing schedule (200 mg every 3 weeks or 400 mg every 6 weeks), and the number of cycles administered. Any dose modifications or treatment delays were also recorded.

Clinical outcomes were captured through follow-up visits and imaging studies. Specifically, information on disease progression or recurrence (including timing and anatomical sites) and treatment-related AEs was systematically collected. AEs were graded according to the *Common Terminology Criteria for Adverse Events (CTCAE), version 4.0.*

*Follow-up time was defined as the interval from the initiation of pembrolizumab plus chemotherapy to the date of the last available clinical or imaging assessment.*

### 2.4 Study endpoints

The study was designed with two primary objectives. The first objective was to comprehensively describe the baseline demographic, clinical, pathological, and biomarker characteristics of patients with stage IV ESCC treated with first-line pembrolizumab plus chemotherapy. This included assessment of age, sex, ECOG performance status, medical comorbidities, presenting symptoms, weight loss, tumor location and morphology, histological grade, T and N stages, metastatic sites, and biomarker status (PD-L1 expression and dMMR/MSI-H).

The second objective was to evaluate real-world outcomes in terms of disease progression and recurrence, including their timing and anatomical distribution, as well as treatment-related toxicities. Adverse events were recorded and graded according to the CTCAE version 4.0, with special attention to both all-grade and Grade ≥3 toxicities and immune-related adverse events (irAEs).

### 2.5 Statistical analysis

Statistical analysis was performed using Stata^®^ 15 (StataCorp LLC, USA). Baseline demographic, clinical, pathological, and biomarker characteristics were summarized using appropriate descriptive statistics. Continuous variables were reported as mean ± standard deviation (SD) when normally distributed or as median with range when skewed. Categorical variables were expressed as absolute numbers and percentages. Patterns of disease progression and recurrence were presented according to frequency, timing, and anatomical distribution. AEs were summarized by type and grade, with results stratified into all-grade and Grade ≥3 categories in accordance with the CTCAE, version 4.0. All statistical analyses were performed using Stata version 17.0 (StataCorp LLC, College Station, TX, USA).

## 3 Results

**Figure 1:**
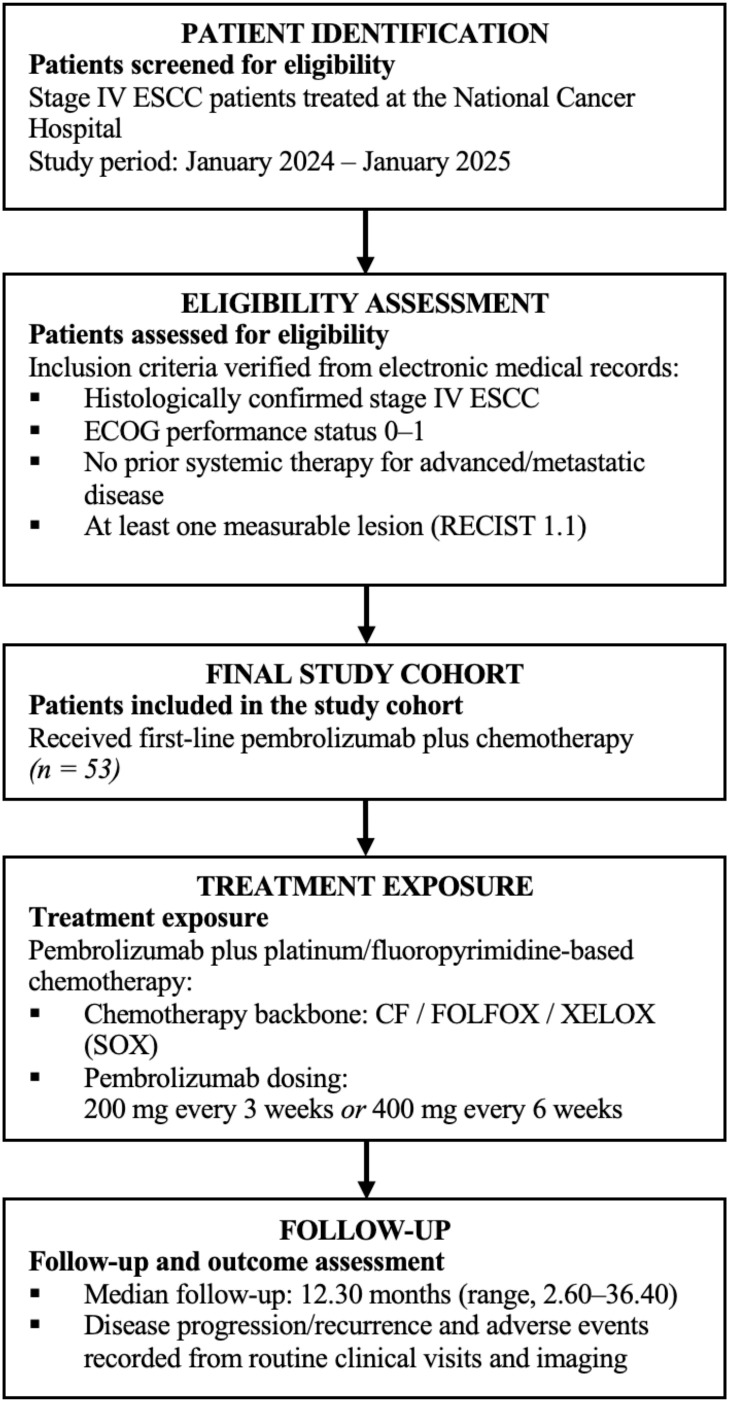
Study flow diagram and treatment exposure.

Patient selection and treatment exposure in this real-world cohort of stage IV esophageal squamous cell carcinoma patients receiving first-line pembrolizumab plus chemotherapy are summarized in **Figure 1**.

With a median follow-up duration of 12.30 months (range, 2.60–36.40 months), the study population comprised 53 patients with stage IV ESCC. The mean age of patients was 59.06 ± 7.36 years, with a range from 47 to 77 years. The majority of patients were male and identified as Kinh ethnicity (98.11%). Regarding performance status, most patients had an ECOG score of 0 (56.60%) or 1 (43.40%). A history of gastric disease was uncommon (5.66%), while 33.96% of patients presented with at least one internal medical comorbidity. A history of drug allergy was rare (1.89%). For PD-L1 expression, 26.42% of patients had a CPS < 1, 20.75% had a CPS between 1 and 10, and 20.75% had a CPS ≥ 10. Thus, 11 patients (20.75%) were classified in the PD-L1 CPS ≥10 category. Information on PD-L1 status was unavailable for approximately 32.08% of patients. dMMR/MSI-H positivity was recorded in 1.89% of the cohort (**Table 1**).

**Table 1:** Baseline demographic and clinical characteristics.

Characteristics	Patients (N = 53)
Age, years	
Mean ± SD	59.06 ± 7.36
Range	47-77
Ethnicity, n (%)	
Kinh	52 (98.11)
Other	1 (1.89)
ECOG Performance Status, n (%)	
0	30 (56.60)
1	23 (43.40)
History of Gastric Disease, n (%)	
Yes	3 (5.66)
No	50 (94.34)
History of Internal Medical Diseases, n (%)	
At least one comorbidity	18 (33.96)
No comorbidities	35 (66.04)
History of Drug Allergy, n (%)	
Yes	1 (1.89)
No	52 (98.11)
Primary Tumor Location, n (%)	
Upper third	13 (24.53)
Middle third	24 (45.28)
Lower third	13 (24.53)
Gastric cardia	1 (1.89)
Unknown	2 (3.77)
Gross Features of Tumor, n (%)	
Mixed	24 (45.28)
Fungating	25 (47.17)
Infiltrating	2 (3.77)
Ulcerative	1 (1.89)
Unknown	1 (1.89)
Histologic Grade, n (%)	
G1 (Well-differentiated)	2 (3.77)
G2 (Moderately differentiated)	19 (35.85)
G3 (Poorly differentiated)	4 (7.55)
Unknown/Not assessed	25 (47.17)
PD-L1 Expression Status (CPS), n (%)	
CPS < 1	14 (26.42)
1 ≤ CPS < 10	11 (20.75)
CPS ≥ 10	11 (20.75)
Not performed/Unknown	17 (32.08)
dMMR/MSI-H Status, n (%)	
Positive	1 (1.89)
Not performed	52 (98.11)
Follow-up duration, months	
Median (range)	12.30 (2.60–36.40)

At the time of diagnosis, dysphagia-related symptoms were the most common presenting complaint (60.38%), followed by lymphadenopathy (20.75%) and routine check-ups/follow-ups (15.09%). The majority of patients experienced weight loss of less than 10% (73.58%). In terms of T stage, T3 was the most prevalent (73.58%), while N2 was the most common N stage (58.49%). The primary tumor was most frequently located in the middle third of the esophagus (45.28%). Gross tumor features were predominantly fungating (47.17%) or mixed (45.28%). Imaging assessments revealed that 30.19% of tumors were confined to the esophagus, and 26.42% showed invasion. Lymph nodes were present in 66.04% of patients. Distant metastasis was identified in 45.28% of patients, with lymph nodes (51.72%) and lungs (20.69%) being the most frequent metastatic sites. Bone scans indicated bone metastasis in 7.55% of patients, and bronchoscopy showed no bronchial invasion in 16.98% of cases (**Table 2**).

**Table 2:** Disease characteristics at diagnosis.

Characteristics	Patients (N = 53)
Presenting Symptoms	
Dysphagia-related symptoms, n (%)	32 (60.38)
Lymphadenopathy, n (%)	11 (20.75)
Routine check-up/Follow-up, n (%)	8 (15.09)
Chest pain/discomfort, n (%)	4 (7.55)
Other symptoms, n (%)	3 (5.66)
Weight Loss, n (%)	
< 10%	39 (73.58)
> 10%	3 (5.66)
No weight loss	11 (20.75)
T Stage, n (%)	
Tis	1 (1.89)
T1b	1 (1.89)
T2	1 (1.89)
T3	39 (73.58)
T4a	8 (15.09)
T4b	1 (1.89)
Unknown	2 (3.77)
N Stage, n (%)	
N0	1 (1.89)
N1	7 (13.21)
N2	31 (58.49)
N3	13 (24.53)
Unknown	1 (1.89)
Metastatic Sites	
Lymph nodes, n (%)	30 (51.72)
Lungs, n (%)	12 (20.69)
Liver, n (%)	6 (10.34)
Bone, n (%)	4 (6.90)
Peritoneum, n (%)	3 (5.17)
Adrenal gland, n (%)	1 (1.72)
Kidney	1 (1.72)
Local Tumor Assessment (Imaging), n (%)	
Confined to esophagus	16 (30.19)
Invasive	14 (26.42)
Not visualized/Not performed	23 (43.40)
Lymph Node Assessment (Imaging), n (%)	
Lymph nodes present	35 (66.04)
No lymph nodes	9 (16.98)
Not performed/Unknown	9 (16.98)
Distant Metastasis Assessment, n (%)	
Metastasis present	24 (45.28)
No metastasis	19 (35.85)
Not performed/Unknown	10 (18.87)
Bone Scan Results, n (%)	
Metastasis present	4 (7.55)
No metastasis	37 (69.81)
Not performed/Unknown	12 (22.64)
Bronchoscopy Results, n (%)	
No invasion	9 (16.98)
Not performed/Unknown	44 (83.02)

The CF (Cisplatin + 5-FU) combination chemotherapy regimen was the most frequently used (50.94%), followed by XELOX/SOX (24.53%) and FOLFOX (18.87%). The Pembrolizumab dose of 200mg every 3 weeks was predominant (69.81%) compared to 400mg every 6 weeks (13.21%). Regarding the number of Pembrolizumab plus chemotherapy cycles, 39.62% of patients received 1–3 cycles, 37.74% received 4–6 cycles, and 15.09% received more than 6 cycles. For Pembrolizumab plus 5FU cycles, 32.08% of patients did not receive any cycles, while 24.53% received 1–3 cycles (**Table 3**).

**Table 3:** Treatment regimen and cycles.

Characteristics	Patients (N = 53)
Chemotherapy Regimen, n (%)	
CF (Cisplatin + 5-FU)	27 (50.94)
FOLFOX	10 (18.87)
XELOX/SOX	13 (24.53)
Unknown	3 (5.66)
Pembrolizumab Dose, n (%)	
200mg/3 weeks	37 (69.81)
400mg/6 weeks	7 (13.21)
Unknown	9 (16.98)
Number of Pembrolizumab + Chemotherapy Cycles, n (%)	
1–3 cycles	21 (39.62)
4–6 cycles	20 (37.74)
> 6 cycles	8 (15.09)
Unknown	4 (7.55)
Number of Pembrolizumab + 5FU Cycles, n (%)	
0 cycles	17 (32.08)
1–3 cycles	13 (24.53)
4–6 cycles	8 (15.09)
> 6 cycles	5 (9.43)
Unknown	10(18.87)

In this cohort, 24.53% of patients experienced disease progression or recurrence during follow-up, with the majority (20.75%) occurring within the first 6 months. The most frequent sites of progression were lymph nodes (11.32%), followed by local recurrence (5.66%) and distant metastases (5.66%) (**Table 4**).

**Table 4:** Progression and recurrence patterns.

Outcome	Value
Progression/Recurrence Status	
Progression/Recurrence present	13 (24.53)
No progression/recurrence	40 (75.47)
Time to Progression/Recurrence	
< 6 months	11 (20.75)
6–12 months	1 (1.89)
> 12 months	1 (1.89)
Site of Progression/Recurrence	
Local	3 (5.66)
Lymph node	6 (11.32)
Distant metastasis	3 (5.66)
No progression/Unknown	41 (77.36)

Hematologic toxicities were common, with anemia (50.94%, Grade ≥3: 1.89%) and neutropenia (43.40%, Grade ≥3: 13.21%) being the most frequent. Febrile neutropenia occurred in 9.43% of patients (Grade ≥3: 1.89%). Gastrointestinal toxicities included nausea (20.75%, Grade ≥3: 1.89%) and diarrhea (5.66%, Grade ≥3: 3.77%). Other treatment-related AEs comprised fatigue/myalgia (11.32%, Grade ≥3: 1.89%), skin rash (7.55%, Grade ≥3: 1.89%), and elevated liver enzymes (15.09%, Grade ≥3: 5.66%). Increased creatinine was observed in 3.77% of patients (no Grade ≥3 events). Immune-related adverse events were less frequent, with hypothyroidism (3.77%) and pneumonitis (3.77%, Grade ≥3: 1.89%) being the main manifestations (**Table 5**).

**Table 5:** Adverse events.

Adverse Event	All Grades (n, %)	Grade ≥ 3 (n, %)
Hematologic Toxicities		
Neutropenia	23 (43.40)	7 (13.21)
Febrile neutropenia	5 (9.43)	1 (1.89)
Anemia	27 (50.94)	1 (1.89)
Thrombocytopenia	3 (5.66)	2 (3.77)
Gastrointestinal Toxicities		
Nausea	11 (20.75)	1 (1.89)
Diarrhea	3 (5.66)	2 (3.77)
Other Treatment-Related AEs		
Fatigue/Myalgia	6 (11.32)	1 (1.89)
Skin rash	4 (7.55)	1 (1.89)
Increased liver enzymes (SGOT/SGPT)	8 (15.09)	3 (5.66)
Increased creatinine	2 (3.77)	0 (0.00)
Immune-Related Adverse Events (irAEs)		
Hypothyroidism	2 (3.77)	0 (0.00)
Pneumonitis	2 (3.77)	1 (1.89)

AEs are presented as frequency and percentage, stratified by all grades and Grade ≥3 according to CTCAE version 4.0.

## 4 Discussion

This study represents the first real-world investigation into the efficacy and safety of first-line pembrolizumab combined with chemotherapy in Vietnamese patients with stage IV ESCC. Our findings provide valuable insights into the clinical characteristics of this specific patient cohort and their outcomes under this contemporary treatment regimen, offering a crucial local perspective that complements data from pivotal global clinical trials.

The baseline characteristics of our cohort largely align with the known epidemiology of ESCC, particularly its higher prevalence in male patients and in Asian populations (4). The mean age of 59.06 years is somewhat younger than cohorts in Western real-world studies, such as Ahn et al. (median age 69 years in 1L cohort) (5), but comparable to the median age of Japanese patients (67.5-68.0 years) in the KEYNOTE-590 subgroup analysis (6). This demographic distinction underscores the importance of regional studies, as age and associated comorbidities can influence treatment tolerability and outcomes. The high rate of dysphagia in our cohort, together with predominance of advanced T3 and N2 disease, reflects the aggressive nature of ESCC and the pattern of late presentation seen in resource-limited settings. Similar findings have been reported in Vietnam, where approximately 87.4% of newly diagnosed esophageal cancer patients experienced dysphagia at presentation (7). This late presentation can significantly impact prognosis and treatment feasibility, necessitating tailored approaches. Notably, a significant proportion of our patients had unknown or unassessed PD-L1 status (32.08%). This reflects practical constraints in routine care, including variable availability of validated assays, specimen limitations and laboratory standardization, which have been reported as barriers to widespread PD-L1 testing outside clinical trials (8).

The prognostic and predictive value of PD-L1 expression has been well established in Asian subgroups of KEYNOTE-181 and other trials, where patients with PD-L1 CPS ≥ 10 derived significantly greater overall survival compared with chemotherapy [KEYNOTE-181 Asian ESCC subgroup: median OS 10.0 vs 6.5 months, HR 0.63; PD-L1 CPS ≥10 cut-offs showing consistent benefit] (2). Meta-analyses further affirm that immunotherapy regimens confer improved survival in ESCC particularly in patients with CPS ≥10 compared to lower PD-L1 expression cohorts (9). However, PD-L1 testing is not uniformly available in Vietnam, and the absence of standardized assays (including issues with CPS assay variability) and reimbursement policies limits its integration into daily practice. Other biomarkers such as mismatch repair deficiency (dMMR)/microsatellite instability-high (MSI-H) are exceedingly rare in ESCC, but some early reports suggest that circulating tumor DNA (ctDNA) dynamics may serve as complementary markers of treatment response. For example, a recent study showed that changes in ctDNA during immunochemotherapy were associated with better tumor regression and may help monitor response in ESCC patients (10). These advances emphasize the need for improved infrastructure to enable biomarker-driven personalization of care in Vietnam.

In our real-world cohort, approximately one-fifth of patients had PD-L1 CPS ≥10, a proportion broadly consistent with that reported in Asian subgroups of pivotal clinical trials. While KEYNOTE-181 demonstrated that patients within this high PD-L1 expression subgroup derive the greatest survival benefit from pembrolizumab, interpretation of PD-L1–stratified outcomes in our study is inherently limited by the single-arm design, the small sample size, and the incomplete availability of PD-L1 testing in routine practice. Moreover, the relatively high proportion of patients with untested PD-L1 status reflects current diagnostic availability and reimbursement constraints in Vietnam, highlighting a gap between evidence-based biomarker stratification and its real-world implementation.

Regarding disease control, our study documented that 24.5% of patients experienced progression or recurrence, with most events occurring within the first six months. Lymph nodes were the most frequent sites of progression, followed by local and distant relapse. These findings are consistent with the aggressive biology of advanced ESCC and mirror recurrence patterns reported elsewhere, where regional lymph node relapse is commonly the predominant mode of failure and recurrence rates are highest within the first years after treatment (11). While pivotal trials such as KEYNOTE-590 have established pembrolizumab plus chemotherapy as a global standard of care, real-world reports from Asia remain limited. Our study contributes locally relevant data from Vietnam, which may complement future multi-center efforts and meta-analyses aimed at clarifying outcomes across diverse Asian populations. The observed progression/recurrence rate of 24.5%, with 20.8% occurring within six months, highlights the difficulty of achieving durable disease control in advanced ESCC. This pattern—early recurrence and frequent involvement of regional lymph nodes (particularly supraclavicular and mediastinal stations)—has been reported in prior series and large cohorts (11).

Although survival endpoints and objective response rates are commonly reported in clinical trials, these outcomes could not be robustly assessed in our study because of the real-world nature of data collection. Radiologic evaluations were performed at non-uniform intervals, and detailed target-lesion measurements required for standardized RECIST-based response assessment were not consistently available. In addition, follow-up duration was heterogeneous and not sufficiently mature to allow reliable estimation of overall survival. Accordingly, our analysis focused on descriptive patterns of disease progression and safety outcomes that could be consistently captured from routine clinical records.

The safety profile observed in our cohort broadly aligns with findings from pivotal trials and real-world series. Hematologic toxicities were common and largely attributable to the chemotherapy backbone; for example, KEYNOTE-590 reported frequent decreases in neutrophil count and anemia with pembrolizumab-chemotherapy combinations (12). Real-world multicenter cohorts from China likewise describe hematologic and gastrointestinal toxicity as among the most frequent AEs and report overall safety outcomes comparable to clinical trials, albeit with some variability across centers and chemotherapy partners (13). Immune-related adverse events (irAEs) were observed but remained less frequent than chemotherapy-related toxicities in our sample. Hypothyroidism and pneumonitis — the two main irAEs in our cohort — are well documented in the literature as characteristic toxicities of PD-1 blockade; pneumonitis in particular has variable incidence and can present early during treatment, requiring prompt recognition and multidisciplinary management (14). Taken together, these consistencies support the generalizability of our safety observations while also highlighting expected differences between controlled trial populations and heterogeneous real-world practice across Asia, where patient selection, supportive care resources, and regimen choice may vary (12).

The safety profile observed in our cohort generally reflects the known adverse event landscape of pembrolizumab combined with chemotherapy, as reported in pivotal trials and real-world studies. Hematologic toxicities, such as neutropenia and anemia, are common; in KEYNOTE-181 (Asia ESCC subgroup), decreased neutrophil count and anemia were among the most frequent any-grade treatment-related AEs, and hypothyroidism and pneumonitis were reported as immune-related AEs (2). Gastrointestinal toxicities such as nausea and diarrhea are expected, and the rates of immune-related toxicities in our study, although lower, mirror those in Asian trials where hypothyroidism (~16-17%) and pneumonitis (~5%) were notable (2). These consistencies help validate our findings despite differences in patient population and real-world practice settings.

This study holds significant value as the first comprehensive real-world analysis of first-line pembrolizumab plus chemotherapy for stage IV ESCC in Vietnam. The data generated directly reflects treatment patterns and outcomes within the Vietnamese healthcare system, offering a crucial local perspective that is often underrepresented in global literature. This local evidence is vital for informing national treatment guidelines, optimizing resource allocation, and tailoring patient management strategies to the specific needs and characteristics of the Vietnamese population. The findings contribute to a more complete understanding of the real-world effectiveness and safety of this regimen in an Asian context, beyond the highly selected populations of clinical trials.

From a practical perspective, the incorporation of pembrolizumab into routine first-line treatment in Vietnam faces significant challenges. The cost of immunotherapy remains a major barrier, with limited coverage from national health insurance schemes, thereby restricting access for many patients. Variability in drug availability between central and provincial hospitals may further exacerbate inequities in care. Another challenge is the recognition and management of immune-related toxicities, which require multidisciplinary collaboration and specialized training that is not uniformly available across institutions. Addressing these issues through policy development, resource allocation, and physician education will be essential to ensure equitable and safe implementation of immunotherapy in Vietnam.

Despite its pioneering nature, this study is subject to several limitations. Firstly, its retrospective, single-center design inherently limits the generalizability of the findings to the broader Vietnamese population and introduces potential for selection bias, as patients treated at a single institution may not fully represent the diversity of ESCC patients across the country. Secondly, the relatively small sample size (N = 53) may limit the statistical power for detecting subtle differences in subgroups and could lead to wider confidence intervals for survival estimates, making it challenging to draw robust conclusions on comparative effectiveness. Thirdly, the lack of a direct comparison arm, such as a historical cohort treated with chemotherapy alone, within this study design prevents definitive conclusions regarding the superiority of the combination regimen in this specific real-world setting. Furthermore, as a retrospective study, it was susceptible to missing data, particularly for certain AEs and detailed response assessments, including individual patient information required for ORR and BOR calculations, which could impact the completeness and precision of the safety and efficacy profiles. The absence of standardized survival and response endpoints also precluded meaningful comparisons with historical chemotherapy-only cohorts. Because the cohort included only patients with ECOG 0–1 who were selected to receive combination immunochemotherapy, the findings may not be generalizable to frailer patients (ECOG ≥2) who are common in routine practice. Information on major lifestyle risk modifiers (e.g., smoking and alcohol use) was incompletely documented in routine practice and could not be included in the analysis. Subgroup analyses according to PD-L1 CPS were limited and descriptive in nature and should therefore be interpreted with caution. Finally, the absence of long-term follow-up data for all patients restricts the ability to report mature overall survival and long-term safety outcomes, which are crucial for assessing the durable benefits of immunotherapy. Future prospective, multi-center studies with larger cohorts and longer follow-up periods are warranted to validate these findings and provide more robust evidence for clinical decision-making in Vietnam.

## 5 Conclusions

This study provides the first real-world evidence on the use of pembrolizumab combined with chemotherapy as first-line treatment for Vietnamese patients with stage IV ESCC. The analysis highlights key demographic and clinical characteristics of this cohort and documents patterns of disease progression and recurrence, as well as the spectrum of treatment-related toxicities. The observed safety profile was generally consistent with international experience, while local challenges such as late presentation and limited biomarker testing were evident. Although restricted by sample size, single-center design, and the absence of mature survival data, these findings offer an important initial reference for clinical practice in Vietnam and emphasize the need for larger, multi-center studies with longer follow-up to further inform treatment strategies for advanced ESCC in this setting.

## Data Availability

The raw data supporting the conclusions of this article will be made available by the authors, without undue reservation.
